# Supplementary material for: Unraveling the Aquaporin-3 Inhibitory Effect of Rottlerin by Experimental and Computational Approaches
Source: Int J Mol Sci. 2023 Mar 22;24(6):6004. doi: 10.3390/ijms24066004 (PMC10057066; doi:10.3390/ijms24066004)
Supplement: Supplementary file 1 [file ijms-24-06004-s001.zip › ijms-2270021-supplementary.pdf]

Unraveling the aquaporin-3 inhibitory effect of rottlerin by experimental and computational approaches

Inês Paccetti-Alves [1,2†], Marta S. P. Batista [3†], Catarina Pimpão [1,2], Bruno L. Victor [3,*] and Graça Soveral [1,2,*]

Supplementary Material

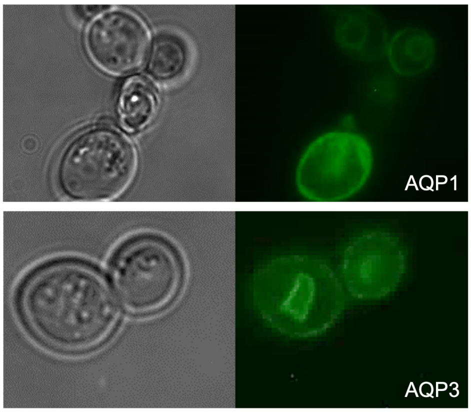

Figure S1 - Localization of GFP-tagged human aquaporins in yeast plasma membrane. Epifluorescence (right panels) and phase contrast (left panels) images of *S. cerevisiae* aqy-null strains transformed with plasmids harboring *hAQP1* and *hAQP3* genes. Cells, grown in YNB medium, show aquaporin localization at the yeast plasma membrane.

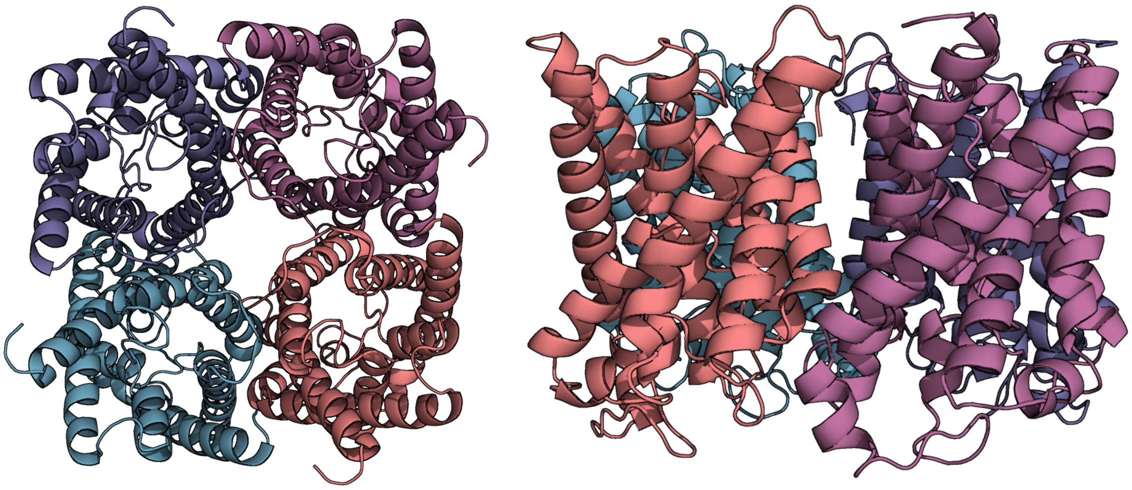

Figure S2 - Structural representation of the tetrameric form of AQP3 generated using comparative modeling.

Unraveling the aquaporin-3 inhibitory effect of rottlerin by experimental and computational approaches

Inês Paccetti-Alves [1,2†], Marta S. P. Batista [3†], Catarina Pimpão [1,2], Bruno L. Victor [3,*] and Graça Soveral [1,2,*]

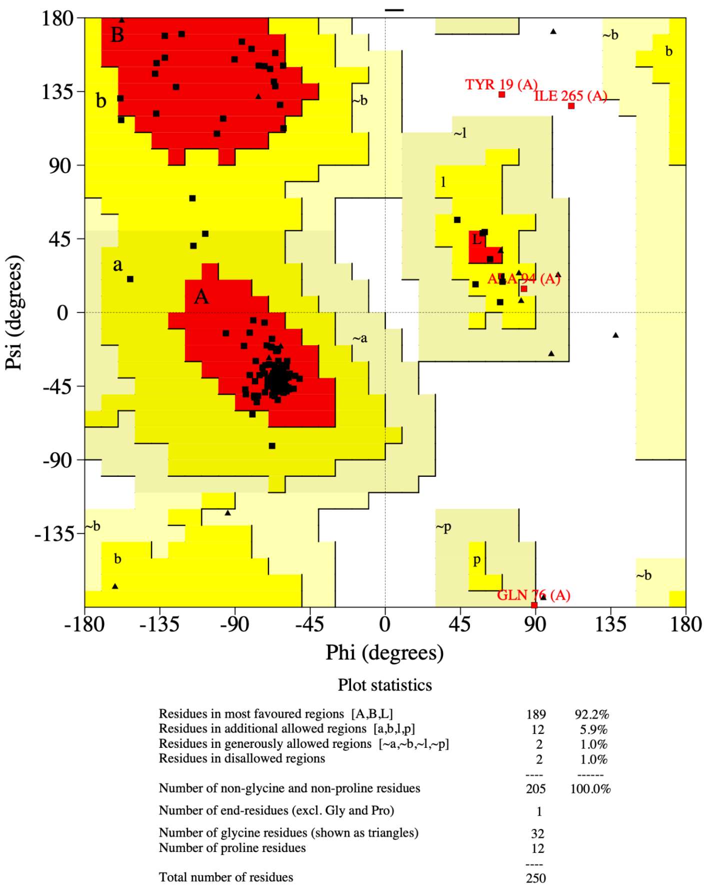

Figure S3 - Ramachandran plot of the best structural model of the monomer of AQP3 protein generated using homology modeling.

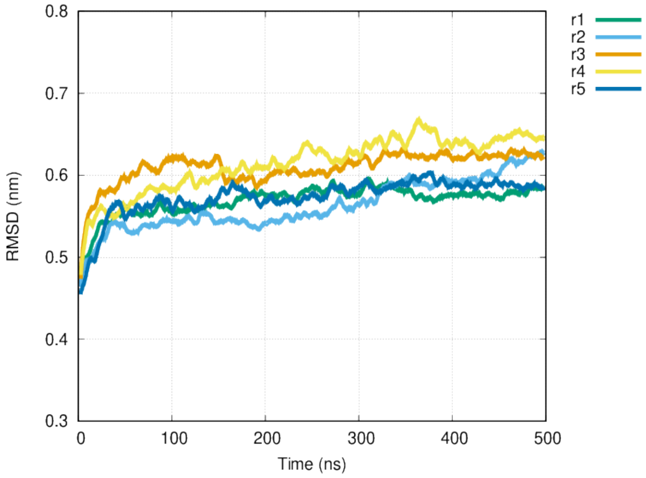

Figure S4 - All atom RMSD plot of the tetrameric structure of AQP3 fully embedded in a POPC model membrane for all replicate MD simulations.

Unraveling the aquaporin-3 inhibitory effect of rottlerin by experimental and computational approaches

Inês Paccetti-Alves [1,2†], Marta S. P. Batista [3†], Catarina Pimpão [1,2], Bruno L. Victor [3,*] and Graça Soveral [1,2,*]

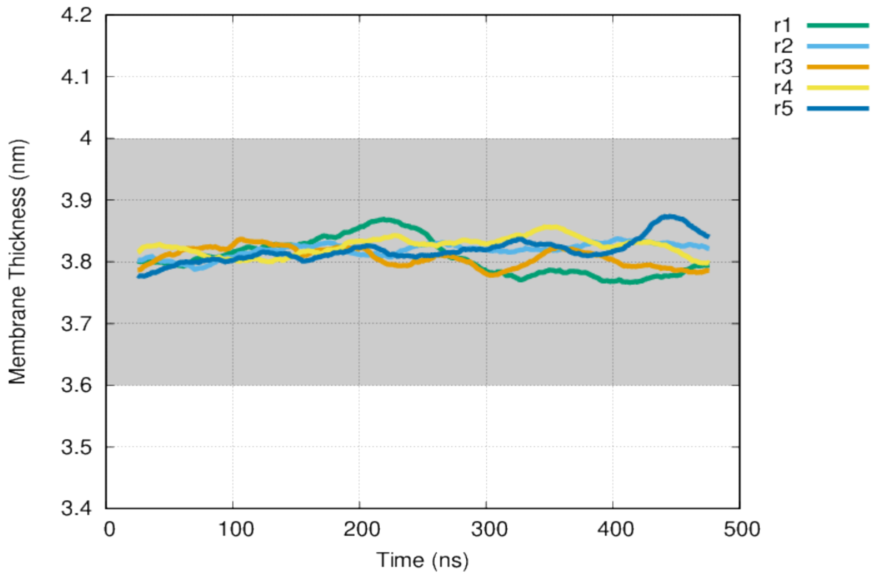

Figure S5 - Average membrane thickness of the lipid bilayer throughout the simulation times for all replicate simulations. The grey shaded area in the plot represents the interval of reported average POPC membrane thickness [44].

Unraveling the aquaporin-3 inhibitory effect of rottlerin by experimental and computational approaches

Inês Paccetti-Alves [1,2†], Marta S. P. Batista [3†], Catarina Pimpão [1,2], Bruno L. Victor [3,*] and Graça Soveral [1,2,*]

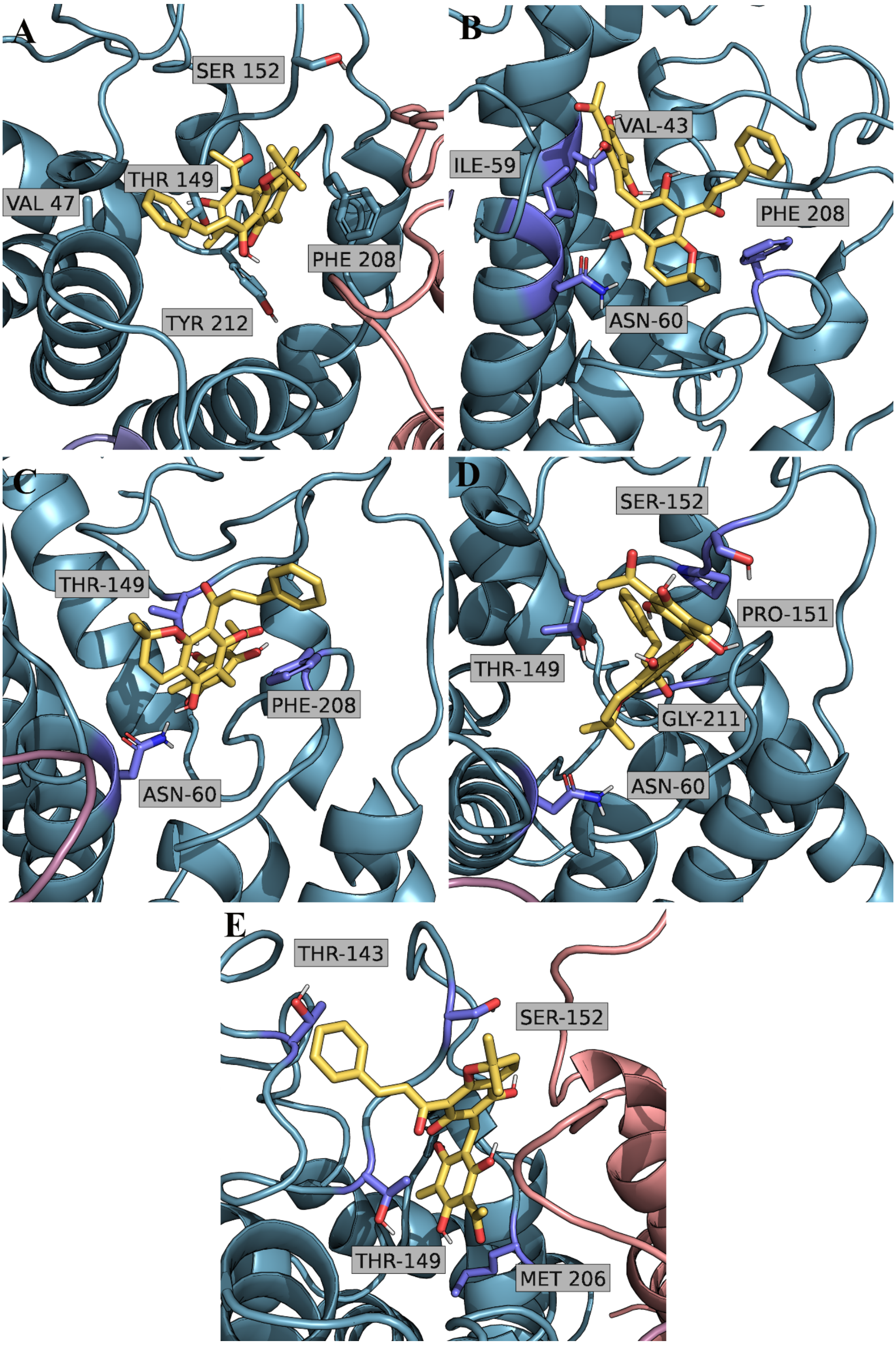

Figure S6 - Representative molecular docking poses of RoT on the binding region of AQP3 taken from the used representative configurations from basin 5.

Unraveling the aquaporin-3 inhibitory effect of rottlerin by experimental and computational approaches

Inês Paccetti-Alves [1,2†], Marta S. P. Batista [3†], Catarina Pimpão [1,2], Bruno L. Victor [3,*] and Graça Soveral [1,2,*]

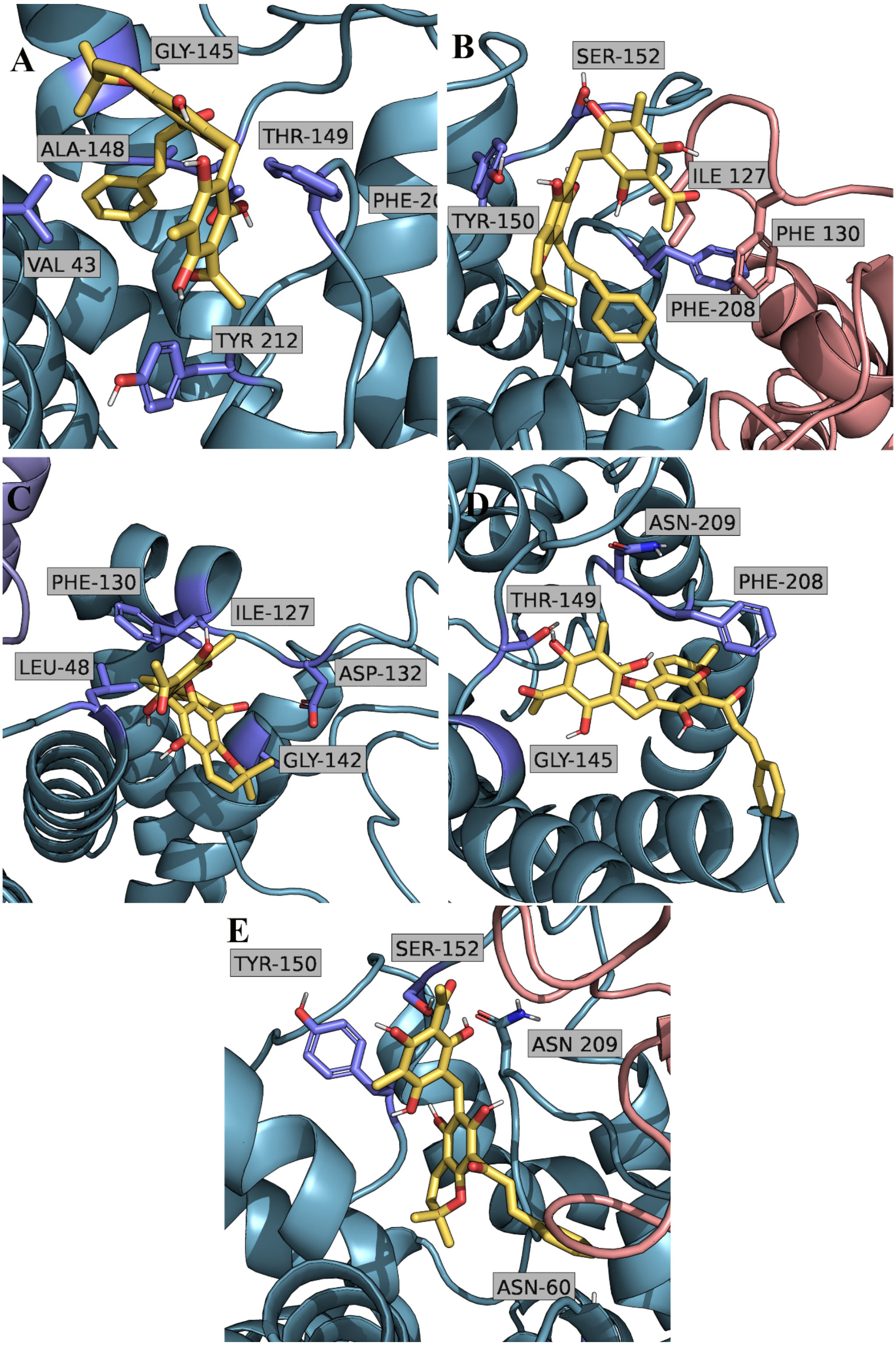

Figure S7 - Representative molecular docking poses of RoT on the binding region of AQP3 taken from the used representative configurations from basin 6.

Unraveling the aquaporin-3 inhibitory effect of rottlerin by experimental and computational approaches

Inês Paccetti-Alves [1,2†], Marta S. P. Batista [3†], Catarina Pimpão [1,2], Bruno L. Victor [3,*] and Graça Soveral [1,2,*]

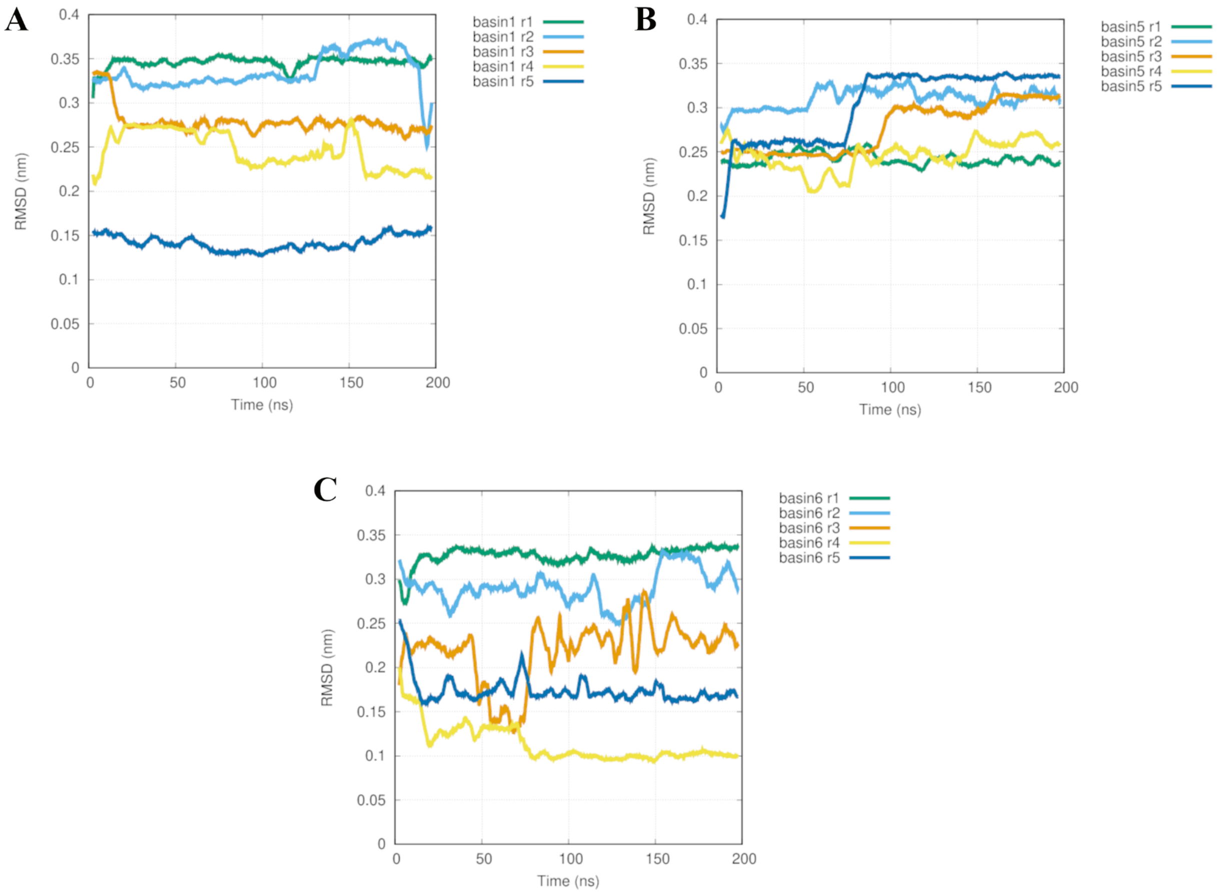

Figure S8 - RMSD profiles of RoT calculated along all replicate MD simulations. The RMSD values were calculated in respect to the best docking pose determined in the molecular docking calculations with AQP3 conformation identified in basin 1, replicate 1.
